# Ascending central canal dilation and progressive ependymal disruption in a contusion model of rodent chronic spinal cord injury

**DOI:** 10.1186/1471-2377-7-30

**Published:** 2007-09-07

**Authors:** Milan Radojicic, Gabriel Nistor, Hans S Keirstead

**Affiliations:** 1 Reeve-Irvine Research Center, Department of Anatomy and Neurobiology, University of California at Irvine, 2111 Gillespie Neuroscience Research Facility, Irvine, CA, 92697-4292, USA; 2 4000 Civic Center Drive, Suite 206, San Rafael, CA 94903, USA

## Abstract

**Background:**

Chronic spinal cord injury (SCI) can lead to an insidious decline in motor and sensory function in individuals even years after the initial injury and is accompanied by a slow and progressive cytoarchitectural destruction. At present, no pathological mechanisms satisfactorily explain the ongoing degeneration.

**Methods:**

Adult female Sprague-Dawley rats were anesthetized laminectomized at T10 and received spinal cord contusion injuries with a force of 250 kilodynes using an Infinite Horizon Impactor. Animals were randomly distributed into 5 groups and killed 1 (n = 4), 28 (n = 4), 120 (n = 4), 450 (n = 5), or 540 (n = 5) days after injury. Morphometric and immunohistochemical studies were then performed on 1 mm block sections, 6 mm cranial and 6 mm caudal to the lesion epicenter. The SPSS 11.5 t test was used to determine differences between quantitative measures.

**Results:**

Here, we document the first report of an ascending central canal dilation and progressive ependymal disruption cranial to the epicenter of injury in a contusion model of chronic SCI, which was characterized by extensive dural fibrosis and intraparenchymal cystic cavitation. Expansion of the central canal lumen beyond a critical diameter corresponded with ependymal cell ciliary loss, an empirically predictable thinning of the ependymal region, and a decrease in cell proliferation in the ependymal region. Large, aneurysmal dilations of the central canal were accompanied by disruptions in the ependymal layer, periependymal edema and gliosis, and destruction of the adjacent neuropil.

**Conclusion:**

Cells of the ependymal region play an important role in CSF homeostasis, cellular signaling and wound repair in the spinal cord. The possible effects of this ascending pathology on ependymal function are discussed. Our studies suggest central canal dilation and ependymal region disruption as steps in the pathogenesis of chronic SCI, identify central canal dilation as a marker of chronic SCI and provide novel targets for therapeutic intervention.

## Background

By the end of the next decade, 300,000 people will be living with chronic spinal cord injury in the US alone [1]. Advances in medical and rehabilitative care have improved survival rates for these individuals, but many experience clinical decline even years after the initial injury. Clinical decline is often accompanied by a slow and progressive cavitation of the central spinal cord, known as post-traumatic syringomyelia (for a review, see [2]). The pathogenesis of this disease remains poorly understood.

Derangements of cerebrospinal fluid (CSF) dynamics following spinal cord injury are thought to play a role in the initiation and propagation of syringomyelic cysts [3-15]. Normally, CSF circulates in the subarachnoid space, traverses the spinal cord several times a day and exhibits a craniocaudal flow pattern influenced by the cardiac cycle [16]. Some spinal fluid, driven by systolic pulsations, is thought to enter the substance of the cord via the Virchow-Robin perivascular spaces and flow toward the central canal [17,18], an enigmatic structure of the central spinal cord believed to function as a CSF pathway. Indeed, Storer and colleagues have suggested that fluid flow through the central canal may comprise a 'sink' function whereby harmful metabolites are removed from the cord [19]. Others believe the spinal cord itself produces extracellular fluid, whose egress toward the subarachnoid space or central canal depends on the pressure differential between the two compartments [16].

Following SCI, normal CSF dynamics may be distorted by a number of possible mechanisms, including subarachnoid CSF outflow obstructions [2,12], changes in compliance of the subarachnoid space [6], or elevated intraspinal pressures [9]. Altered CSF dynamics are believed to result in localized spinal cord edema, known as the presyrinx state [20] that subsequently gives rise to intraspinal cavities and cysts.

The propagation of intraspinal cavities requires a driving force sufficient to propel fluid via a one-way valve mechanism into the cysts and cavities, which often contain fluid at a higher pressure than the subarachnoid space [21]. Proposed driving forces include cardiac pulsations along vessels [4,17], postural changes and valsalva movements [22], coalescence of microcysts [23] and elevated intraspinal pressures [9]. We would additionally suggest consideration of the transient hypertensive episodes of autonomic dysreflexia (for a review, see [24,25]) and the oncotic pressure of central canal fluid as potential driving forces.

The histological features of syringomyelia have been reproduced in experimental models of the disease. Experimental syringomyelia was first induced reliably with intraspinal injections of the irritant kaolin, producing histological results similar to syringomyelia and, in many respects, acute hydrocephalus [14]. Subsequent models utilized intraspinal injections of excitotoxic compounds and produced pathological results via a physiologically relevant mechanism consistent with the neurochemical milieu known to accompany SCI [5,26,27]. Both experimental models suffer the limitation of being non-traumatic [26] and require the administration of exogenous agents [28]. Mathematical models of syrinx formation allow the study of pressure wave propagations in an idealized cerebrospinal fluid system, but suffer the limitation of not taking into account spinal cord parenchymal changes that may influence patterns of dilation [3,4,8-10,13,29].

Here, we utilized a clinically relevant contusion model of chronic SCI in hopes of elucidating novel features of this insidious disease. Reminiscent of human syringomyelia, chronic spinal cord injured animals developed cysts and glial-lined cavities of the central spinal cord near the epicenter of injury, which was characterized by extensive dural fibrosis. A prominent ascending and progressive dilation of the central canal lumen cranial to the epicenter of injury was evident over time, presumably due to distensile forces within the canal, and was accompanied by changes in the surrounding ependyma and periependymal tissues. By Laplace's law, we reasoned that dilations in the central canal lumen would result in a wall tension being exerted on the ependymal cells lining the canal that would increase with both the radius and pressure within the canal. We therefore focused our study on the histopathological changes in the ependymal region and periependymal tissues that accompanied the progressive dilation of the central canal.

## Methods

### Spinal cord injury

Adult female Sprague-Dawley rats (n = 22) aged 6–8 weeks and weighing 200–220 grams were anesthetized with intraperitoneal injections of Ketamine (80 mg/kg; Phoenix Pharmaceuticals, Inc. St. Joseph, MO) and Xylazine (10 mg/kg; Phoenix Pharmaceuticals, Inc., St. Joseph, MO). The skin overlying the T8-T11 vertebral levels was shaved and disinfected with provodone scrubs. An incision was made over the T8-T11 spinous processes exposing the underlying paravertebral muscles. Blunt dissection was utilized to expose the transverse processes at T9-T11, and a complete laminectomy was performed at T10. The spinous processes of T9 and T11 were clamped and stabilized using a stereotactic device and a contusion injury with a force of 250 kilodynes was induced using an Infinite Horizon Impactor (Precision Systems and Instrumentation LLC, Fairfax, VA). The overlying muscle was then sutured in layers and the skin stapled with stainless steel wound clips. Post-operative care included bolus injections of subcutaneous saline and prophylactic enrofloxacin (2.5 mg/kg/day; Bayer Corporation, Shawnee Mission, KS). Animals received manual bladder expression twice daily and were inspected for signs of infection, dehydration or autophagia, with appropriate veterinary assistance as needed. All surgical interventions and pre- and post-operative care were conducted in accordance with institutional guidelines.

### Histology

Animals were randomly distributed into 5 groups and killed 1 (n = 4), 28 (n = 4), 120 (n = 4), 450 (n = 5), or 540 (n = 5) days after injury. Animals in the 1, 28, 120 and 450-day groups were terminally anesthetized and killed by intracardiac perfusion with 4% glutaraldehyde (Fisher Scientific International Inc., Pittsburgh, PA) for resin processing. Animals in the 540-day group received intraperitoneal injections of bromodeoxyuridine (BrdU, 100 mg/kg; Sigma-Aldrich, St. Louis, MO) daily over a two week period prior to terminal anesthesia and killing by intracardiac perfusion with 4% paraformaldehyde for immunohistochemical processing; these spinal cords was dissected and cut in the transverse plane into twelve 1 mm blocks, six blocks cranial to and six blocks caudal to the lesion epicenter. Spinal cords from the 1, 28, 120 and 450-day groups were post-fixed in 4% glutaraldehyde for 72 hours, rinsed in 0.1 M phosphate buffer pH 7.4 for 30 minutes, exposed to 1% OsO4 (Electron Microscopy Sciences, Fort Washington, PA), dehydrated in ascending alcohols, soaked in propylene oxide (Electron Microscopy Sciences, Fort Washington, PA), and embedded in Spurr resin (Electron Microscopy Sciences, Fort Washington, PA), as previously described [30,31]. Transverse semi-thin (1 um) sections were cut from the cranial face of each block, stained with alkaline toluidine blue (Sigma, St. Louis, MO), cover slipped and examined by light microscopy on an Olympus AX-80 microscope using Olympus Microsuite B3SV software (Olympus America Inc., Melville, NY). For electron microscopy, blocks were trimmed and sections were cut at 100 nm, mounted on copper grids, stained with uranyl acetate and lead citrate, and viewed under a Hitachi EM 600 electron microscope at 75 kV (Hitachi High Technologies America Inc., Schaumburg, IL). For the 540-day group, tissue blocks were cut with a cryostat into 20 μm sections and mounted on gelatin-coated slides for BrdU immunohistochemistry and hematoxylin/eosin staining (Sigma-Aldrich, St. Louis, MO).

For BrdU immunostaining, slide mounted sections from the 540 day group were denatured for 30 minutes using 2 N HCL (Fisher Scientific, Hampton, NH) and then incubated for 30 minutes in 0.1 M phosphate buffer pH 7.4 with 2% bovine serum albumin (Sigma-Aldrich, St. Louis, MO). Sections were incubated overnight at 4 degrees Celsius with a mouse monoclonal anti-BrdU immunoglobulin (Zymed Laboratories, San Francisco, CA). Bound antibody was visualized by incubation in goat anti-mouse Alexa Fluor 594 secondary antibody (Invitrogen, Carlsbad, CA) for 1 hour. Finally, slides were coverslipped and viewed using an Olympus AX-80 microscope using Olympus Microsuite B3SV software (Olympus America Inc., Melville, NY). Standard immunohistochemical controls were utilized and untreated sections and no-primary antibody controls were observed for evidence of autofluorescense.

Sections adjacent to those used for BrdU immunostaining were stained with hematoxylin and eosin according to standard protocol [32]. Hematoxylin/eosin stained sections were observed to verify the presence and characteristics of ependymal region nuclei and to rule out misidentification of infiltrating autofluoresecent cells such as macrophages.

### Quantitative analysis of the ependymal region

Five transverse sections sampled at successive 1 mm increments cranial and caudal to the lesion epicenter were analyzed from each animal. Sections were excluded from analyses in cases where secondary non-ependymal lined cavities disrupted the integrity of the central canal. Sections were viewed and digitally photographed with an Olympus AX-80 microscope using Olympus Microsuite B3SV software (Olympus America Inc., Melville, NY). Ependymal region area, central canal luminal area, central canal perimeter and ependymal region nuclear count measurements were made using the Olympus Microsuite B3SV software (Olympus America Inc., Melville, NY). Only clearly stained, round to oval nuclei were counted. Average ependymal region thickness was calculated as the average of eight linear measurements, taken at 8 compass points, from the apical-most ependymal cell processes in contact with the central canal lumen to the basal-most ependymal cell process in contact with the basal lamina of the ependymal region. The ependymal region cellular density was determined by dividing the total ependymal region nuclear count by the corresponding central canal luminal perimeter. Cellular coverage of the central canal was determined by multiplying ependymal region cellular density by the diameter of an average ependymal cell. The SPSS 11.5 t test was used to determine differences between quantitative measures.

## Results

**Figure 1:**
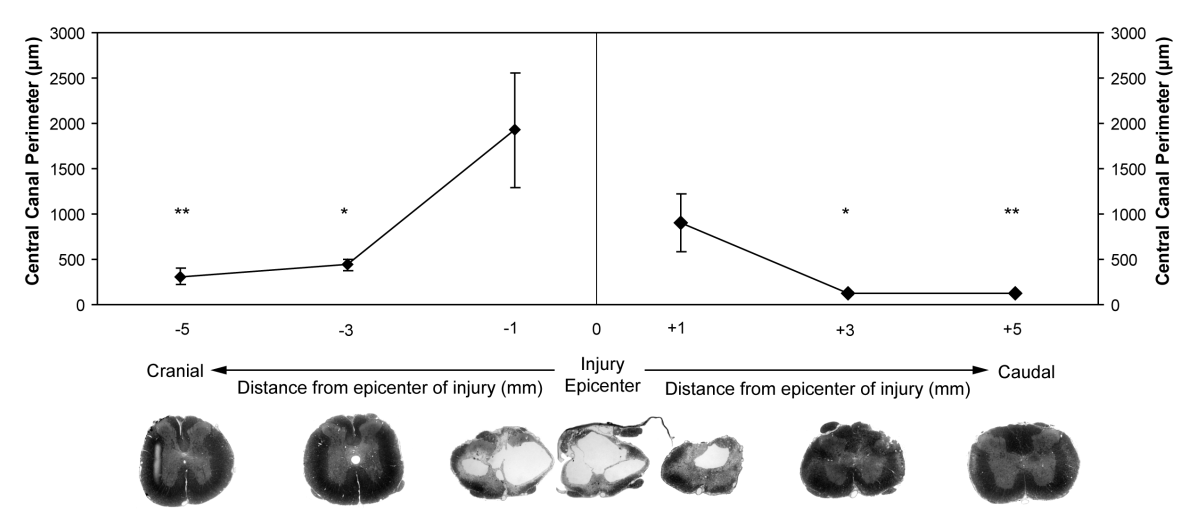
Quantification of the central canal perimeter +/- 1 mm-5 mm from the lesion epicenter 450 days after injury, with representative toluidine blue stained transverse sections of rat spinal cord for each distance. Extensive dural fibrosis and intraparenchymal cavitations are evident at the epicenter of the injury, which are reminiscent of human syringomyelia. Central canal dilation is more marked cranial to the epicenter of injury and is evident up to 5 mm cranial to epicenter of the injury. Asterisks indicate significance at p < 0.05. Magnification = 10×.

By 450 days after spinal cord injury, all rats developed cysts and glial-lined cavitations of the central spinal cord near the epicenter of injury, which was characterized by extensive dural fibrosis. An ascending dilation of central canal lumen was noted up to 5 mm cranial to the injury epicenter that was not evident to this extent caudal to the injury (Figure 1). A statistically significant (p < 0.05) difference in central canal perimeter was noted between the distances 3 mm cranial and 3 mm caudal to the lesion epicenter, as well as 5 mm cranial and 5 mm caudal the lesion epicenter, compared to normal, uninjured spinal cords at the same vertebral level.

**Figure 2:**
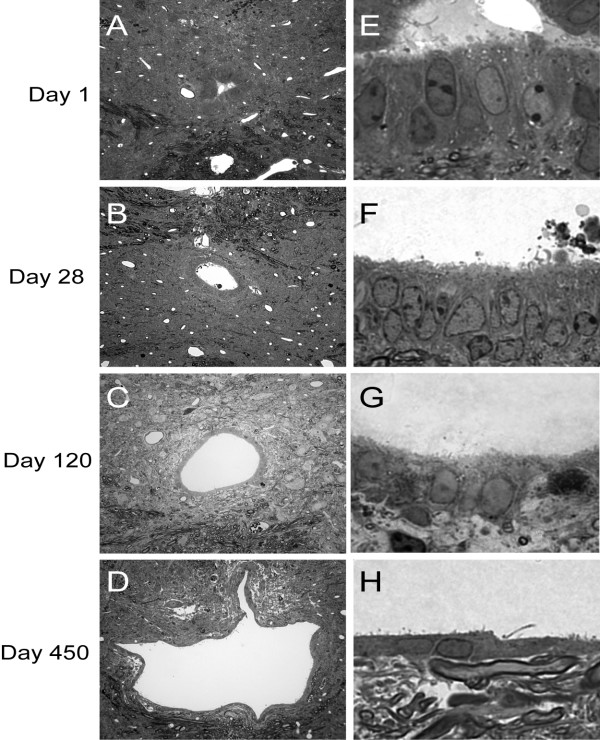
Qualitative changes over time of the post-traumatic central canal lumen, ependymal and periependymal regions at 1 mm cranial to the lesion epicenter. Toluidine blue stained transverse sections of contused rat spinal cords 1 mm cranial to the lesion epicenter are shown in **A-H**. Low magnification images reveal a progressively increasing central canal lumen from day 1 (**A**), day 28 (**B**), day 120 (**C**) and day 450 (**D**) following injury. High magnification images reveal progressive thinning of the ependymal region, ependymal ciliary loss, and periependymal edema from day 1 (**E**), day 28 (**F**), day 120 (**G**) and day 450 (**H**) following injury. Magnification = 40× for A-D, 300× for E-H.

**Figure 3:**
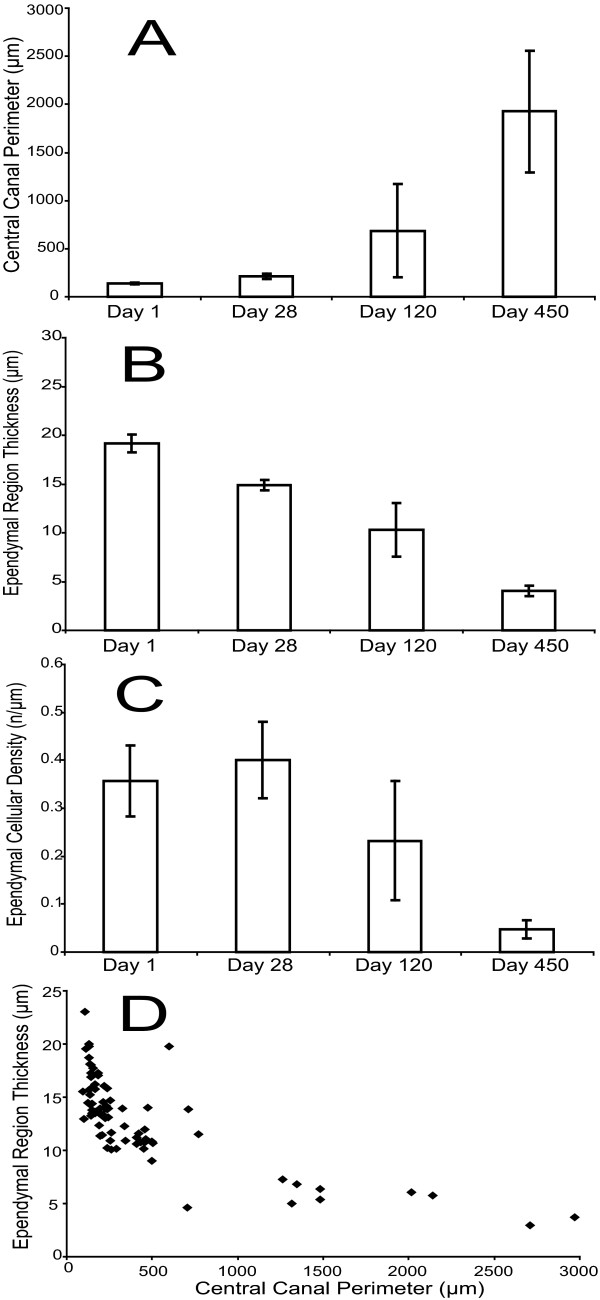
Quantitative changes over time of the post-traumatic central canal lumen, ependymal and periependymal regions at 1 mm cranial to the lesion epicenter. (**A**) Quantitation of central canal perimeter 1 mm cranial to the lesion epicenter indicates a progressive increase from day 1 to day 450 following injury. (**B**) Quantitation of ependymal region thickness 1 mm cranial to the lesion epicenter indicates a progressive decrease from day 1 to day 450 following injury. (**C**) Quantitation of ependymal region nuclear density 1 mm cranial to the lesion epicenter indicates an increase from day 1 to day 28 following injury, then a progressive decrease from day 28 to day 450 following injury. (**D**) The relation of ependymal region thickness to perimeter of the central canal lumen at all time points. Error bars represent standard error.

The development of central cystic cavitations at the epicenter of injury was accompanied by qualitative and quantitative trends in the central canal rostral to the epicenter of injury (Figure 2, Figure 3). Changes were noted in central canal luminal perimeter, the ependymal region thickness and periependymal tissue consistency. Toluidine blue staining revealed progressive dilation of the central canal lumen immediately (1 mm) cranial to epicenter of injury over time (Figure 2A–D), accompanied by progressive thinning of the ependymal region, ependymal cell ciliary loss as well as periependymal edema, gliosis and destruction of the surrounding neuropil (Figure 2E–H). Quantitative analysis of these changes over time revealed a progressive increase in dilation of the central canal lumen (Figure 3A), a progressive decrease in the average ependymal region thickness (Figure 3B), and a progressive decrease in the average ependymal region cellular density (Figure 3C). There was a statistically significant increase in the total ependymal region nuclear count from day 1 to day 28 (data not shown), which was reflected in an increasing cellular density from day 1 to day 28, but this latter measure did not achieve statistical significance. A comparison of central canal perimeter and ependymal region cellular density at 1 mm cranial to the lesion epicenter for time points 1 and 28 days versus day 450 indicated a statistically significant (p < 0.05) negative correlation. Pooling of data from all time points and distances rostral to the lesion epicenter indicated that the thickness of the ependymal region varied inversely with the size of the central canal (Figure 3D).

**Figure 4:**
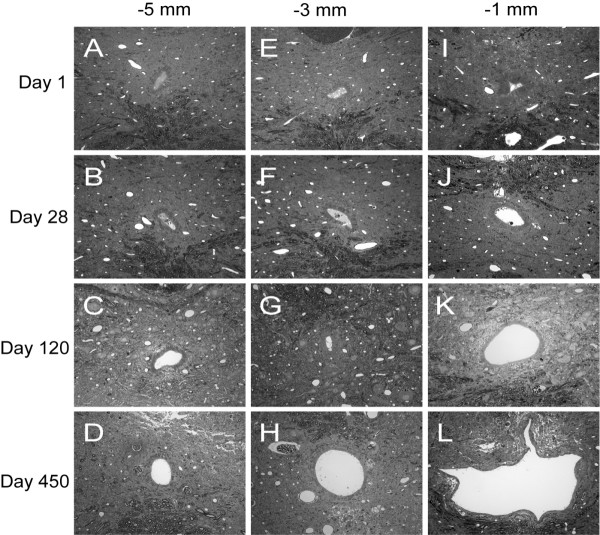
Qualitative changes over time of the post-traumatic central canal lumen and ependymal region cellular density at 1 mm-5 mm cranial to the lesion epicenter. Toluidine blue stained transverse sections of contused rat spinal cords from day 1, day 28, day 120 and day 450 following injury at 1 mm (**A-D**), 3 mm (**E-H**) and 5 mm (**I-L**) cranial to the lesion epicenter. Magnification = 40× for A-L.

**Figure 5:**
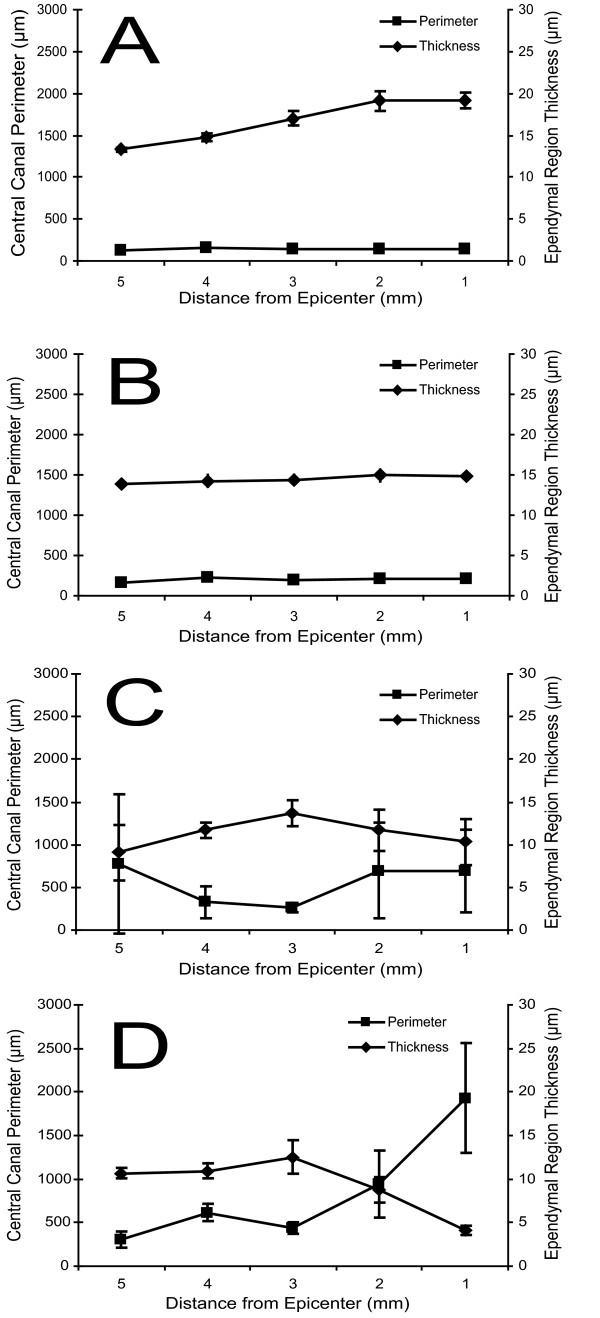
Quantitative changes over time of the post-traumatic central canal lumen and ependymal region cellular density at 1 mm-5 mm cranial to the lesion epicenter. Quantitation of central canal perimeter and ependymal region thickness from 1 mm-5 mm cranial to the lesion epicenter at day 1 (**A**), day 28 (**B**), day 120 (**C**), and day 450 (**D**) following injury. Error bars represent standard error.

Changes over time in the central canal lumen and ependymal region cellular density were also evident in the region of spinal cord extending 1–5 mm cranial to the lesion epicenter. Enlargement of the central canal lumen from 1 to 450 days post-injury was evident at 1 mm (Figure 4A–D), 3 mm (Figure 4E–H) and 5 mm (Figure 4I–L) from the lesion epicenter; the increase in central canal lumen size was greater closer to the lesion epicenter and, at late time points, spread progressively to rostral spinal cord segments. Quantitative analysis of central canal lumen perimeter and ependymal region cellular density indicated relatively stable values from 1–5 mm cranial to the lesion epicenter at 1 day (Figure 5A) and 28 days (Figure 5B) post injury. By day 120 post-injury (Figure 5C), marked and varied dilations of the central canal lumen perimeter were evident at all points cranial to the lesion epicenter. At 450 days post-injury (Figure 5D), the central canal lumen was markedly dilated 1 mm cranial to the epicenter of the injury and was accompanied by diminishing dilations of the central canal lumen in adjacent spinal cord segments up to 5 mm cranial to the epicenter of injury. Notably, an inverse relationship between central canal size and ependymal region thickness was evident over the 1 to 3 mm distance at 450 days, similar to the trend seen at 1 mm over time.

**Figure 6:**
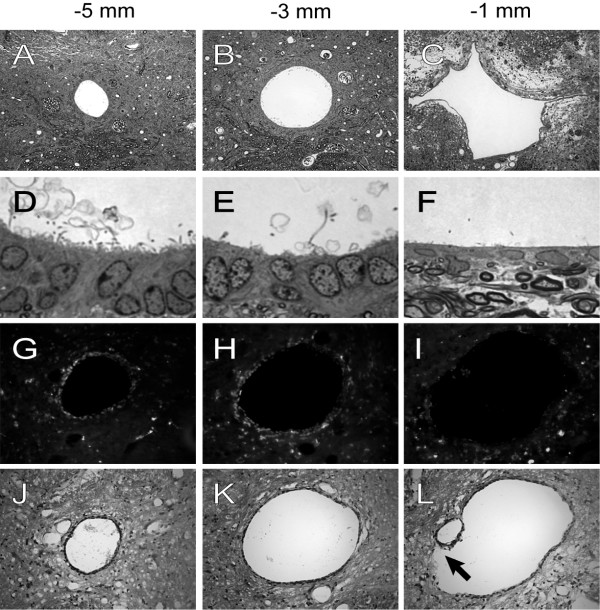
Transverse sections of the adult rat spinal cord at late time points after injury. Toluidine blue staining 450 days after injury 5 mm (**A**), 3 mm (**B**), and 1 mm (**C**) from the lesion epicenter reveals a progressive dilation of the central canal. Higher power magnification of the cells lining the central canal 5 mm (**D**), 3 mm (**E**), and 1 mm (**F**) from the lesion epicenter reveals a progressive loss of cilia and flattening of ependymal cells at 1 mm from the lesion epicenter (**F**); these cells also exhibit a more euchromatic appearing nucleus. (**G**) BrdU immunolabeling of the central canal at 540 days after injury 5 mm from lesion epicenter reveals BrdU immunoreactivity with a mild dilation of the central canal. (**H**) BrdU immunolabeling of the central canal 3 mm from lesion epicenter also reveals BrdU immunoreactivity with a moderate dilation of the central canal, but with more dispersion of cells around the canal. (**I**) BrdU immunolabeling of the central canal 1 mm from lesion epicenter shows diminished BrdU reactivity with severe dilation of the central canal. Hematoxylin and eosin staining at 540 days after injury 5 mm (**J**), 3 mm (**K**), and 1 mm (**L**) from the lesion epicenter reveals a progressive dilation of the central canal; note that the continuity of cells bordering the central canal is disrupted 1 mm from the lesion epicenter (arrow in **L**). Magnification = 40× for **A&B**, 30× for **C**, 300× for **D**-**F**, 40× for **G**-**L**.

By 450 days post-injury and beyond, marked dilations of the central canal lumen perimeter were evident at all points cranial to the lesion epicenter, with a progressive increase in the central canal lumen perimeter toward the lesion epicenter (Figure 6A–C). Associated with the progressive increase in central canal lumen perimeter toward the lesion epicenter was a progressive loss of ependymal cilia and thinning of the ependymal region (Figure 6E–F). In order to assess the effects of these structural changes in the ependymal region cells on the proliferative capacity of ependymal region cells, BrdU was administered. A decrease in BrdU immunostaining was found within the ependymal region that accompanied large, aneurysmal dilations of the central canal lumen (Figure 6G–I). Serial sections adjacent to the BrdU immunostained sections were hematoxylin and eosin stained (Figure 6J–L), revealing discontinuity of the ependymal layer surrounding the large, aneurysmal dilations of the central canal at 1 mm cranial to the lesion epicenter (arrow in Figure 6L).

**Figure 7:**
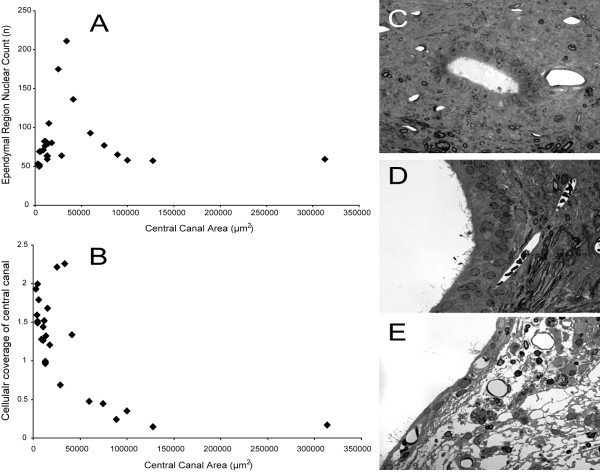
The progressive dilation of the central canal with time after spinal cord injury results in a loss of ependymal region cellular density. (**A**) Total ependymal region cell counts increase with enlarging central canal area up until a critical point. Thereafter, the absolute cell number decreases. (**B**) Cellular coverage of the central canal similarly decreases with enlarging central canal. Transverse toluidine blue stained sections of the adult rat spinal cord 450 days after injury demonstrating the evolution of the ependymal region cell changes are depicted in **C-E**. Mild (**C**) and moderate (**D**) central canal dilations are associated with an increasing ependymal region nuclear count. Large, aneurysmal dilation of the central canal (**E**) is accompanied by flattening of the ependymal cell layer, ependymal ciliary loss and disruptions in the continuity of cells lining the canal. Note the periependymal edema and gliosis, macrophage infiltration and loss of adjacent neuropil. Magnification = 60× for **C-E**.

Comparison of the central canal luminal area with the absolute number of cells within the ependymal region revealed a bell-shaped distribution, where the absolute number of cells within the ependymal region increased with enlarging central canal area up until a critical area of approximately 40,000 μm^2 ^(corresponding to a diameter of approximately 220 μm); thereafter, the absolute cell number decreased (Figure 7A). While the absolute nuclear count returned to pre-peak values, plotting the extent of cellular coverage of the central canal cell revealed an incomplete cellular coverage of the canal (<1 cell) with diameters greater than 220 um (Figure 7B). Transverse toluidine blue stained sections of the adult rat spinal cord 450 days after injury demonstrated that mild (Figure 7C) and moderate (Figure 7D) dilations of the central canal were consistently associated with an ependymal region that was several nuclear layers thick that increased up to a critical diameter; thereafter, the large, aneurysmal dilations of the central canal (Figure 7E) were consistently associated with an ependymal region that displayed a flattened ependymal cell layer, ependymal ciliary loss and a disrupted continuity of cells lining the canal. In all cases, such ependymal regions were juxtaposed to a neuropil with robust degeneration, including edema, gliosis, and macrophage infiltration.

## Discussion

The development of successful therapeutic interventions for chronic spinal cord injury necessitates a greater understanding of the pathogenesis of the disease, including the delineation of its histopathological stages. Here, we describe and provide a temporal framework for several histopathological stages of SCI disease progression.

Spinal cord injured rats demonstrated an ascending and progressive dilation of the central canal lumen cranial to the lesion epicenter over time, which was associated with progressive degeneration of the ependymal and peri-ependymal regions. The central canal is an enigmatic structure of the central spinal cord, believed to function as a CSF pathway. Central canal dilation has been noted in autopsy studies of syringomyelic patients [33] and an ultrastructural analysis of post-traumatic syrinxes revealed ependymal remnants lining portions of the cavity [34]. Using serial MR imaging, Takamura and colleagues have documented a case of post-traumatic progressive central canal dilation leading to syrinx formation in a young adult [35], but until now this feature has not been fully appreciated in an experimental model of the disease. In our studies, mild to moderate dilations of the central canal lumen were not associated with gross degeneration of the ependymal and peri-ependymal regions, whereas large aneurysmal dilations of the central canal lumen were consistently associated with a circumferential degeneration of the ependymal and peri-ependymal regions, indicating that gray matter degeneration in chronic SCI is preceded by changes in the ependymal region and/or intraluminal cerebrospinal fluid (CSF) dynamics. This pathogenic sequence is consistent with that seen in development of chronic hydrocephalus and suggests that distensile forces within fluid compartments resulting from obstruction may be common feature of the two diseases. Furthermore, the central canal dilation we observed was ascending and asymmetric from the point of injury suggesting a pathologic process beyond the mere loss of volume of surrounding tissue. In spinal cord injury, this ascending pathology may represent a tertiary form of injury arising from disturbances in CSF flow near the epicenter of injury arising from dural fibrosis. Moreover, the slow and progressive nature of the phenomenon may result from transient spikes in hydrostatic pressure, leading to a water hammer effect, and/or changes in intraluminal oncotic pressures that would precede ischemic changes in the cord. In humans, the cystic and cavitary lesions of syringomyelia are known to progress over time and thus the transmission of distensile pressures along the central canal may represent a mechanism by which these lesions can spread to adjacent, uninjured spinal cord segments. Our rodent data indicate that the progression occurred cranial to the lesion epicenter, whereas in humans progression occurs both cranial and caudal to the lesion epicenter. This difference may be influenced by the effects of gravity on the upright posture of humans.

Spinal cord injured rats demonstrated progressive ependymal cell ciliary loss cranial to the lesion epicenter, and a loss of ependymal cell cilia with large dilations of the central canal lumen. Cilia are specialized projections of ependymal cells that promote the flow of CSF within the central canal. Ependymal ciliary loss is known to predispose to hydrocephalus[36] and is a feature of the disease [37]. Therefore, this loss of ependymal cell cilia in the spinal cord may result in altered local CSF homeostasis, resulting in an accumulation of toxic metabolites and oncotic pressures, which could result in damage to the ependymal and periependymal regions. Notably, the ependymal cell layer disruption noted in our study is reminiscent of the ependymal denudation that proceeds the development of severe hydrocephalus in the hyh mouse [38].

At late time points, large aneurysmal dilations of the central canal lumen were accompanied by denudation of the ependymal cell layer. Normally, ependymal cells form a pseudostratified monolayer of epithelium that regulates fluid and electrolyte balance between the CSF and neuropil [39]. Disruption of the ependymal layer could therefore result in the loss of a protective epithelium. This loss of integrity and competence of the canal could result in exposure of the adjacent grey and white matter to hydrostatic and oncotic pressure gradients, leading to a dissection of stagnant CSF from within the canal into the gray and white matter of the cord, leading to structural and conductive deficits. Indeed, fluid from syringomyelia cysts is known to differ from normal CSF [40], usually having a higher protein content [41] and prolonged exposure of peri-ependymal tissues to this microenvironment may lead to its degeneration. In our studies, areas of ependymal denudation were consistently opposed to regions of peri-ependymal edema, gliosis, macrophage infiltration and loss of neuropil. Furthermore, local disruption of the blood brain barrier may contribute to the edema [40] and subsequent ischemic injury, but may also represent a source of inflammatory molecules and plasma proteins that may adversely influence the microenvironment of nearby cells, including cells with stem/progenitor characteristics, thereby influencing their viability and patterns of differentiation.

It is intriguing to note that large, aneurysmal dilations of the central canal lumen were also associated with decreased proliferation of ependymal region cells. Cells of the ependymal region are vestiges of neuroepithelial cells that give rise to neurons and glia during mammalian development [42] and are known to orchestrate the regenerative response in tailed amphibians [43]. Ependymal region cells have been shown to proliferate [42,44-46] and migrate [47-49] following spinal cord injury. This finding has led some authors to speculate on their role in endogenous repair in humans [50]. Indeed, the kinetics of ependymal region cell proliferation and differentiation have been correlated with the recovery of lower limb motor function in rats following contusion injuries [46]. Of note, neural stem cells have been isolated from the CNS [47,51,52], including regions near the central canal [53]. Unlike the subventricular zone, the prototypical stem cell niche of the CNS (for a review, see [54]), multipotent cells of the ependymal region appear restricted to glial lineages [46,47,49]. Gliogenesis near the central canal includes the generation of ependymal cells [44], reactive astrocytes [46,47,49], oligodendrocyte precursors [55] and microglia [56]. Glia are supportive cells of the CNS and are critical for maintaining the structural and functional integrity of the spinal cord after injury [57]. Even reactive astrocytes, long thought to be inhibitory to axonal regeneration, appear to play a role in repair of SCI lesions [46,58,59]. Therefore, it stands to reason that disruption of the ependymal stromal epithelium, along with periependymal stem/progenitor cells, may represent a heretofore unrecognized pathogenic mechanism in spinal cord injury, which would hinder gliogenesis in the ependymal region and subsequently wound repair in the spinal cord. Indeed, the progressive disruption of this cell layer, through mechanical and cytotoxic means, could represent a disease mechanism that tips the balance between injury and repair in the spinal cord toward further cytoarchitectural destruction of lesions over time. In principle, this conceptualized disease process should be investigated in other multipotent niches as a basis for understanding related degenerative disorders and sequelae.

## Conclusion

This study documents an ascending and progressive central canal dilation and ependymal region destruction rostral to the epicenter of injury in a contusion model of SCI, which was characterized by extensive dural fibrosis near the epicenter of injury. We suggest progressive ependymal region disruption as a novel step in the pathogenesis of chronic spinal cord injury, identify central canal dilation as a potential marker of chronic spinal cord injury and document the ependymal region as a therapeutic target, through cellular rescue or replacement. Our findings share many histological features with chronic hydrocephalus [37] and suggest that early restoration of favorable CSF hydrodynamics in SCI may prevent further degeneration and provide an environment more hospitable for repair.

## Competing interests

The author(s) declare that they have no competing interests.

## Authors' contributions

MR conceived of the pathophysiologic hypotheses and experimental design, participated in the histologic studies, carried out the data analysis and figure formation and drafted the manuscript. GN participated in the design of the chronic SCI experimental model, carried out the resin-based histologic studies and participated in the data analysis. HSK conceived of the chronic SCI experimental model and participated in its design and coordination. All authors read and approved the final manuscript.

## Pre-publication history

The pre-publication history for this paper can be accessed here:


